# Maternal genetic liability for neuroticism and breastfeeding intention, initiation and maintenance

**DOI:** 10.1192/bjo.2026.12046

**Published:** 2026-07-14

**Authors:** Elizabeth C. Braithwaite, Amy Campbell, Holly Fraser, Nicky Wright, Jasmine Hearn, Jennifer McGahan, Lucy Bowes, Alex S.F. Kwong, Hannah Sallis, Rebecca M. Pearson

**Affiliations:** School of Psychology, Manchester Metropolitan University, UK; School of Psychological Science, University of Bristol, UK; Medical Research Council (MRC) Integrative Epidemiology Unit, University of Bristol, UK; Centre for Academic Mental Health, University of Bristol, Bristol; Department of Experimental Psychology, University of Oxford, UK; Centre for Clinical Brain Sciences, Division of Reproductive and Developmental Sciences, University of Edinburgh, UK

**Keywords:** Neuroticism, breastfeeding, maternal mental health, ALSPAC

## Abstract

**Background:**

Relationships between maternal mental health and breastfeeding outcomes are complex, with evidence of bi-directional effects complicated by culturally specific residual confounding and selection bias.

**Aims:**

This study aimed to examine, for the first time, associations between maternal genetic liability for neuroticism and breastfeeding intention, initiation and maintenance.

**Method:**

We used data from the Avon Longitudinal Study of Parents and Children (ALSPAC) prospective birth cohort. Maternal genotype data were used to create polygenic scores (PGS) for neuroticism. Mothers self-reported breastfeeding intentions at 32 weeks gestation, breastfeeding initiation within the first week after birth and maintenance of breastfeeding to 6 months after birth. We examined associations between maternal genetic liability for neuroticism and breastfeeding outcomes by using unadjusted logistic regression models (*n* = 4611). We also examined associations between paternal genetic liability for neuroticism and breastfeeding outcomes as a negative control (*n* = 1446).

**Results:**

There was evidence that genetic liability for neuroticism was associated with lower odds for breastfeeding maintenance (odds ratio 0.86, 95% CI 0.83–0.94, *p* < 0.001), with no evidence for associations with intention or initiation. That is, mothers with higher genetic liability for neuroticism were less likely to maintain breastfeeding to 6 months postpartum. There were no associations between paternal PGS for neuroticism and breastfeeding outcomes.

**Conclusions:**

The specificity of links between neuroticism and maintenance of breastfeeding could direct targeting of efforts to support women with emotional difficulties in maintaining breastfeeding. This is important because currently, most efforts to support breastfeeding women focus on intention and initiation.

The World Health Organization (WHO) recommend that infants are exclusively breastfed for the first 6 months of life,^1^ and then are breastfed alongside solid food for 2 years and beyond. Abundant research has therefore sought to understand why some women are successful at establishing and maintaining breastfeeding and others are not, so that breastfeeding support services can be appropriately targeted.^2–4^ A focused area of research on this topic is the complex relationship between poor mental health in the postnatal period – in particular, experiences of postnatal depression and anxiety – and breastfeeding outcomes. Women with postnatal depression are less likely to breastfeed their child,^5^ and new research demonstrates that depression during pregnancy^6^ and even preceding conception^7^ is associated with poorer breastfeeding outcomes. Women with postnatal depression are more likely to report lower breastfeeding self-efficacy,^8,9^ more worries about breastfeeding^10^ and more breastfeeding difficulties.^11^ However, this literature is limited by small samples of participants with symptoms of depression,^10,11^ and omission of potential confounders from statistical analyses.^8^

Although much of the research has focused on postnatal depression, the broader phenotype and transdiagnostic factor neuroticism (the propensity to feel and experience negative emotions^12^), which is robustly associated with increased risk for depression across adolescence,^13^ and early adulthood for postnatal depression,^14^ is also associated with poor breastfeeding outcomes. For example, mothers with high neuroticism traits are less likely to intend to breastfeed,^15^ have lower breastfeeding self-efficacy^16^ and a lower likelihood of maintaining breastfeeding once established.^17^ However, this literature is also limited by small sample sizes^15^ and the omission of potential confounding variables from the statistical analyses.^16,17^ If neuroticism is linked with breastfeeding, then the neuroticism phenotype potentially has greater utility than the depression phenotype for identifying those at risk of poor breastfeeding outcomes, as depression is episodic, whereas neuroticism is a stable trait.^18^ It is important to consider that engagement in breastfeeding has also been associated with improvements in postnatal mental health,^19^ whereas breastfeeding challenges are associated with poor mental health,^20^ so the relationship between breastfeeding and maternal mental health is unlikely to be unidirectional and linear. It is not clear, therefore, whether an existing propensity for negative emotionality may make breastfeeding more difficult, leading to early cessation, or whether difficulties with breastfeeding may lead to the new onset of, or worsen existing, mental health symptoms. It is likely that to some extent, both directions of effect are evident; however, it is still important to understand whether neuroticism is causally linked to breastfeeding patterns, so that appropriate support services can be targeted, regardless of any subsequent experience of breastfeeding having an impact on later depression.

The relationship between breastfeeding and maternal mental health is further complicated by confounding variables that are culturally specific.^21^ For example, in Western populations, the intention, initiation and maintenance of breastfeeding is strongly influenced by maternal education,^22,23^ age^24^ and parity,^24^ which are also associated with neuroticism traits.^25–27^ This additional complexity adds to the difficulty of understanding exactly how maternal mental health and breastfeeding behaviours are related to each other. In the UK, breastfeeding is widely promoted as the optimal feeding method, but despite this, breastfeeding rates in the UK are very low, whereby only 1% of mothers exclusively breastfeed to 6 months after birth.^28,29^

In the current study, we used a genetic epidemiology design with UK data to examine the associations between maternal genetic liability for neuroticism and breastfeeding intention, initiation and maintenance. The use of this methodology advances current knowledge in two important ways. First, because an individual’s genotypes are randomly inherited and determined at conception, they cannot be influenced by later experiences, in this case breastfeeding. Thus, the study design allows us to understand, in part, the directionality of the relationship between maternal mental health (using genetic liability for neuroticism as a proxy^14^) and breastfeeding outcomes, because the genetic liability for neuroticism traits was determined in each participant before their later experiences of breastfeeding. We can therefore investigate whether a potential causal association exists between the propensity to experience negative emotions and poor breastfeeding outcomes. Second, existing research is limited by social and cultural confounding structures related to both breastfeeding behaviours and maternal mental health.^21^ However, because we are examining genetic liability for neuroticism and not the trait itself, residual confounding is minimised. We hypothesised that a high maternal polygenic score (PGS) for neuroticism liability would be associated with reduced intention to breastfeed, as well as lower likelihood of breastfeeding initiation and maintenance of breastfeeding to 6 months after birth. We additionally examined associations between paternal PGS for neuroticism and breastfeeding outcomes as a negative control. This is because mothers and fathers share many of the same of confounding and unmeasured characteristics that might link to neuroticism (such as living circumstances, educational level and attitudes toward health), but the proposed causal mechanism of neuroticism influencing psychological confidence/capacity to continue breastfeeding is maternal-specific. We therefore hypothesised no association between paternal PGS for neuroticism and breastfeeding outcomes.

## Method

### Participants

The sample comprised participants from the Avon Longitudinal Study of Parents and Children (ALSPAC), an ongoing population-based study. Pregnant women resident in Avon, UK, with expected dates of delivery between 1 April 1991 and 31 December 1992 were invited to take part in the study. A total of 20 248 pregnancies were identified as being eligible and the initial number of pregnancies enrolled was 14 541. Of the initial pregnancies, there was a total of 14 676 fetuses, resulting in 14 062 live births and 13 988 children who were alive at 1 year of age. When the oldest children were approximately 7 years of age, an attempt was made to bolster the initial sample with eligible cases who had failed to join the study originally. As a result, when considering variables collected from the age of 7 onward (and potentially abstracted from obstetric notes), there are data available for more than the 14 541 pregnancies mentioned above. The number of new pregnancies not in the initial sample (known as phase 1 enrolment) that are currently represented in the released data and reflecting enrolment status at 24 years of age is 906, resulting in an additional 913 children being enrolled (456, 262 and 195 recruited during phases 2, 3 and 4, respectively). The phases of enrolment are described in more detail in the cohort profile paper and its update.^30–32^ The total sample size for analyses using any data collected after 7 years of age is therefore 15 447 pregnancies, resulting in 15 658 fetuses. Of these, 14 901 children were alive at 1 year of age. Of the original 14 541 initial pregnancies, 338 were from women who had already enrolled with a previous pregnancy, meaning 14 203 unique mothers were initially enrolled in the study. As a result of the additional phases of recruitment, a further 630 women who did not enrol originally have provided data since their child was 7 years of age. This provides a total of 14 833 unique women (G0 mothers) enrolled in ALSPAC as of September 2021.^33^ Please note that the study website contains details of all the data that is available through a fully searchable data dictionary and variable search tool (http://www.bristol.ac.uk/alspac/researchers/our-data).

The authors assert that all procedures contributing to this work comply with the ethical standards of the relevant national and institutional committees on human experimentation and with the Helsinki Declaration of 1975, as revised in 2013. Ethical approval for the study was obtained from the ALSPAC Ethics and Law Committee and the Local Research Ethics Committees. Consent for biological samples has been collected in accordance with the Human Tissue Act (2004). Informed consent for the use of data collected via questionnaires and clinics was obtained from participants following the recommendations of the ALSPAC Ethics and Law Committee at the time. ALSPAC participants were included in the current study if they had provided genetic and relevant breastfeeding phenotypic data.

### Measures

#### PGS for neuroticism

A total of 116 independent variants were found to be robustly associated with neuroticism by Luciano et al.^34^ Of these original variants, 109 were available in ALSPAC (ALSPAC was not included in this genome-wide association study). The neuroticism PGS variable was created using PRSice-2,^35^ by weighting the effect sizes of the single-nucleotide polymorphisms (SNPs) associated with neuroticism from the initial GWAS at nine *p*-value thresholds (5 × 10−08, 5 × 10−07, 5 × 10−06, 5 × 10−05, 0.0005, 0.005, 0.05, 0.5 and 1) (see Supplementary Table 1). The neuroticism PGS were standardised to have a mean of 0 and a standard deviation of 1; thus, a higher PGS represents higher genetic liability to neuroticism. We included SNPs that had a minor allele frequency of >1% and info score of >80%, and excluded SNPs with an *R* ^2^ of >0.1 if they were within 250 kb of each other. This was to account for linkage disequilibrium so that only the most strongly associated SNPs from each region were retained. Complete genotyping information is available in the Supplementary Materials.

#### Breastfeeding measures

Breastfeeding intention was assessed with a questionnaire administered at 32 weeks gestation. Mothers were asked how they intended to feed their child in the first week. This variable was dichotomised, with mothers who intended to breastfeed exclusively or mix breast and bottle in one category (yes, did intend to breastfeed), and mothers who intended to bottle-feed only or were uncertain in the other category (no, did not intend to breastfeed). ALSPAC variable names related to the breastfeeding items used in this analysis are reported in Supplementary Table 2.

Breastfeeding initiation was measured at 4 weeks postpartum. Mothers were asked how they fed their baby in the first 24 h, and first week. Mothers who answered that they had breastfed exclusively or mixed breast and bottle in either the first 24 h or first week were included in one category (yes, did initiate breastfeeding), and mothers who used bottles exclusively or answered ‘other’ were included in the other category (no, did not initiate breastfeeding).

Breastfeeding maintenance was measured at 6 months postpartum. Mothers were asked if they continued to breastfeed their baby to 6 months after birth. A dichotomous variable was created, so that mothers who responded that they were still breastfeeding were in one category (yes, maintained breastfeeding), and those who had not breastfed or had stopped before 6 months were in the other (no, did not maintain breastfeeding).

### Statistical analyses

We examined the demographic characteristics of the mothers with complete genetic data and the whole ALSPAC sample. Next, we examined the descriptive statistics of the breastfeeding intention, initiation and maintenance variables (number and percentage of binary outcomes: yes/no). In the main analyses, the breastfeeding intention, initiation and maintenance variables were regressed onto the maternal neuroticism PGS via unadjusted logistic regression modelling. Our main analyses were conducted at the *p* = 0.05 PGS threshold score because this explained the most variance, and we also conducted a sensitivity analysis with different PGS threshold scores. We then repeated the main analysis with the paternal neuroticism PGS in place of the maternal score. All analyses were conducted in Stata (version 17.0, Stata Corp LLC, Texas, USA; www.stata.com).

## Results

### Demographics and descriptive statistics

**Fig. 1:**
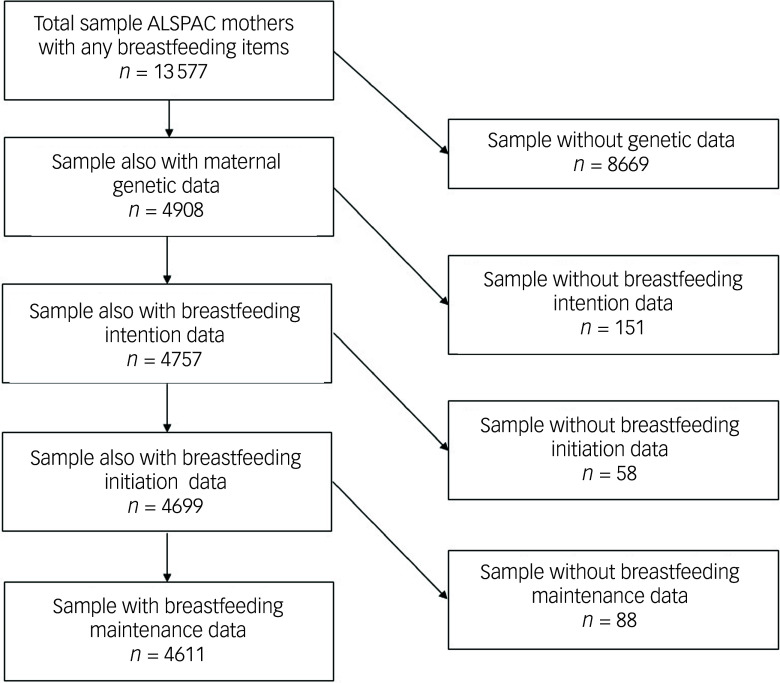
Flowchart to demonstrate the selection of sample for data analysis. ALSPAC, Avon Longitudinal Study of Parents and Children.

A total of *N* = 13 577 ALSPAC G0 mothers had any breastfeeding data available, and of these, *n* = 4908 mothers also had genetic data available. Demographic characteristics for the mothers from the whole ALSPAC sample, and those who had genetic data available for analysis are displayed in Table 1. Participants with missing breastfeeding intention data (*n* = 151), initiation data (*n* = 58) and maintenance data (*n* = 88) were removed, resulting in a final analysis sample of *n* = 4611. A flowchart demonstrating how we arrived at the final sample for analysis is shown in Fig. 1.

**Table 1:** Descriptive data for mothers for whom complete genetic data were available and were included in analyses, and those with missing genetic data

Variable	Whole ALSPAC sample		Sample with genetic data	
	*n*	%	*n*	%
Highest educational qualification				
General Certificate of Secondary Education (GCSE)	2524	20.11	575	12.46
Vocational	1229	9.79	371	8.04
O-level	4319	34.42	1589	34.44
A-level	2792	22.25	1258	27.26
University degree	1606	12.80	809	17.53
Parity				
Zero	5864	43.97	2146	46.19
One	4576	34.31	1628	35.04
Two or more	2661	21.72	825	17.76
Ever smoked				
Yes	6733	50.49	2073	44.62
No	6493	48.69	2553	54.95

ALSPAC, Avon Longitudinal Study of Parents and Children.

The descriptive statistics of the breastfeeding intention, initiation and maintenance variables are shown in Supplementary Table 3. A total of 76.83% of the participants in the sample reported during pregnancy that they intended to breastfeed, and 74.29% of the participants initiated breastfeeding within the first week after the birth of their child. However, just 32.23% of the sample maintained breastfeeding to 6 months after birth.

### Breastfeeding intention, initiation and maintenance

In the models displayed in Table 2, maternal PGS for neuroticism was not associated with either breastfeeding intention during pregnancy (odds ratio 0.97, 95% CI 0.91–1.04, *p* = 0.445) or breastfeeding initiation within the first week of birth (odds ratio 0.98, 95% CI 0.91–1.04, *p* = 0.476). However, maternal PGS for neuroticism was associated with lower odds of breastfeeding maintenance to 6 months after birth (odds ratio 0.86, 95% CI 0.81–0.92, *p* < 0.001). This means that those mothers with a higher genetic liability for neuroticism, i.e. the propensity to experience negative emotions, were less likely to maintain breastfeeding to 6 months after birth than mothers with a low genetic liability for neuroticism. Patterns of association were similar when using different thresholds for the PGS neuroticism score (see Supplementary Table 4).

**Table 2:** Unadjusted models examining the relationship between maternal polygenic scores for neuroticism and breastfeeding intention, initiation and maintenance

Model	Outcome variable	Odds ratio	s.e.	*p*-value	95% CI
*n* = 4611	Breastfeeding intention	0.972	0.035	0.445	0.906–1.044
Pseudo *R* ^2^< 0.001					
*n* = 4611	Breastfeeding initiation	0.975	0.034	0.476	0.911–1.044
Pseudo *R* ^2^< 0.001					
*n* = 4611	Breastfeeding maintenance	0.864	0.027	<0.001	0.813–918
Pseudo *R* ^2^ = 0.003					

Paternal PGS for neuroticism was not associated with maternal breastfeeding intention, initiation or maintenance (see Table 3 for results).

**Table 3:** Unadjusted models examining the relationship between paternal polygenic scores for neuroticism and maternal breastfeeding intention, initiation and maintenance

Model	Outcome variable	Odds ratio	s.e.	*p*-value	95% CI
*n* = 1446	Breastfeeding intention	0.927	0.076	0.353	0.789–1.088
Pseudo *R* ^2^< 0.001					
*n* = 1446	Breastfeeding initiation	0.918	0.073	0.283	0.785–1.073
Pseudo *R* ^2^= 0.001					
*n* = 1446	Breastfeeding maintenance	0.977	0.053	0.972	0.878–1.087
Pseudo *R* ^2^ = 0.003					

## Discussion

This is the first study to examine whether maternal PGS for neuroticism was associated with several breastfeeding outcomes: intention to breastfeed measured during pregnancy, breastfeeding initiation within the first week of birth and breastfeeding maintenance to 6 months postpartum. We hypothesised that an increased maternal PGS for neuroticism would be associated with a lower likelihood of intention to breastfeed, breastfeeding initiation and breastfeeding maintenance. Our hypotheses were partly supported. In unadjusted logistic regression analyses, we found that maternal PGS for neuroticism was not associated with either breastfeeding intention or breastfeeding initiation, but it was associated with breastfeeding maintenance. That is, mothers with an increased genetic liability for neuroticism were less likely to maintain breastfeeding to 6 months postpartum than mothers who had a lower genetic liability for neuroticism. Critically, this indicates that maternal neuroticism may be associated with reduced persistence with breastfeeding, but not with initial behavioural decisions to breastfeed. In the current study, the use of maternal PGS for neuroticism as a proxy for poor maternal mental health advances existing knowledge in the field in two important ways. First, it allowed us to potentially examine directionality of the impact of maternal mental health on breastfeeding outcomes, and second, to minimise the impact of residual confounding.

Despite the knowledge that neuroticism is a key transdiagnostic factor across unipolar mood disorders, very little research has considered the relationship between neuroticism and breastfeeding. Neuroticism represents one of the most robust personality-based risk factors for parental postnatal depression,^14,36^ and its impact in the perinatal period is often mediated by coping strategies^37^ and attachment-related vulnerabilities.^38^ The limited research that has examined the relationship between neuroticism and breastfeeding has been highly consistent with the postnatal depression literature: maternal neuroticism traits are associated with reduced intentions to breastfeed,^15^ lower breastfeeding self-efficacy^16^ and reduced likelihood of breastfeeding maintenance.^17^ These findings, however, are limited by cross-sectional study designs where maternal reports of breastfeeding outcomes and mental health are obtained concurrently. In our study, genetic liability for neuroticism, as a proxy for maternal mental health, was determined at conception, so this study design allows us to examine directionality of effects. Our findings therefore shed light on associations between genetic liability for neuroticism and breastfeeding outcomes, highlighting that genetic liability for neuroticism may not be associated with intentions to breastfeed or breastfeeding initiation in the first week after birth, but is rather associated with a reduced likelihood of breastfeeding maintenance to 6 months postpartum, in line with WHO guidance.^1^ This is important because 6 months of exclusive breastfeeding is associated with reduced risk of gastrointestinal and respiratory infection in infants, compared with 3–4 months of exclusive breastfeeding followed by supplements with formula and/or solid food.^39^ The association between maternal genetic liability for neuroticism and breastfeeding maintenance could be explained by the fact that poor mental health in the perinatal period is specifically associated with more self-reported breastfeeding difficulties.^6,7^ It is therefore possible that women with a higher genetic liability for neuroticism may experience more difficulties with breastfeeding, leading to early cessation. However, this association needs to be directly tested in future work. Additionally, we know that there is a relationship between neuroticism and interpersonal relationships. For example, neuroticism is positively associated with rejection sensitivity^40^ and relationship dissatisfaction.^40^ Therefore, it may be that women with high neuroticism traits are both more likely to experience breastfeeding difficulties, and more likely to feel rejected by their infant and dissatisfied with their experiences of breastfeeding in this context.

The finding of no association between paternal PGS for neuroticism and infant feeding outcomes is supportive of the idea that maternal emotionality is critical to the maintenance of breastfeeding. However, it should be noted that the smaller sample available for the analysis of the partner data may not have been sufficiently powered to detect statistically significant effects, so these results should be interpreted with caution. Further research is needed to delineate the exact mechanism by which genetic liability for neuroticism may result in early breastfeeding cessation, and this is an important research question. Nonetheless, this finding has important clinical implications because currently, healthcare professionals are very focused on promoting the benefits of breastfeeding to mothers during pregnancy (with the aim of increasing breastfeeding intention) and supporting mothers to establish the initiation of breastfeeding within the first few weeks after birth. This is evident in the current study, where 78.86% of mothers intended to breastfeed, and breastfeeding intention was very strongly associated with breastfeeding initiation (odds ratio 37.13). This demonstrates the utility of promoting breastfeeding intention because of its clear impact on breastfeeding initiation. However, there is little support from healthcare professionals for mothers in the UK with the maintenance of breastfeeding past this point.^41^ Our findings therefore highlight the need for continued breastfeeding support once breastfeeding has been initiated. Additionally, mothers who experience a high degree of negative emotionality during this time may be a particularly vulnerable group that need specific support with breastfeeding maintenance.

The use of genetic scores allows new insight into the potential direction of the association because we know the genetic liability for neuroticism came before the experience of breastfeeding. That said, this does not mean we fully understand the pathway from genetic propensity to behaviour.^42^ A limitation of using PGS for neuroticism is that they explain only a small proportion of the variance in the neuroticism trait (approximately 10%^43^), and how the PGS links to observed traits is complex. Although there is a correlation with later measures of mental health,^13,44,45^ including the measured phenotype of neuroticism (rather than the PGS) would be problematic. This approach would introduce bias, as it was measured after breastfeeding (and therefore could be influenced by experiences of breastfeeding or related potential consequences of higher neuroticism) and has measurement error. Another potential limitation of using PGS to investigate causal pathways relates to horizontal pleiotropy, i.e. the action of one genetic variant on multiple unrelated phenotypes. Horizontal pleiotropy is a particular problem when looking at psychiatric traits, which are highly polygenic, and the biological function of the associated genetic variants are often unknown.^46,47^ This limits our ability to draw causal conclusions about direct effects between the neuroticism PGS and breastfeeding outcomes, as this association could operate via one or more other phenotypes. Additionally, participants from the ALSPAC cohort with available genetic data differed on several prenatal demographic characteristics than the sample of participants who did not provide genetic data, which might introduce collider bias if those characteristics are associated with both exposure and outcome. However, given the direction of drop-out, this would be predicted to result in an underestimate of the association between PGS for neuroticism and breastfeeding. For example, participants who have higher neuroticism traits and do not maintain breastfeeding are more likely to drop out, and these are the participants with the association of interest. The driver of missing data were having genetic data, and we are unable to impute genetic data, and so cannot estimate the extent of this potential bias directly; but as outlined above, it is not likely to explain the higher association with PGS for neuroticism and breastfeeding maintenance. If anything, it has diluted that effect. Another consideration is that these data were collected in 1991/1992, over 30 years ago, and a limitation of how the breastfeeding data were collected at 6 months is that participants were only asked about duration of breastfeeding, not whether they were exclusively breastfeeding or a combination of breastfeeding with formula. Additionally, the use of binary breastfeeding variables may have resulted in the loss of some important information. Guidance on breastfeeding in the UK has changed very little during this time, but there has been an intense period of public health messaging around the ‘breast is best’ message, resulting in high rates of breastfeeding expectation.^48^ However, rates of breastfeeding intention, initiation and maintenance to 6 months have changed very little in the UK since the G0 ALSPAC data were collected.^28,49^ Strengths of this study include the genetic epidemiology design and longitudinal nature of the data collection.

In conclusion, we advance existing knowledge on associations between maternal mental health and breastfeeding outcomes in two important and novel ways. First, the use of genetic scores allows new insight into the potential direction of the association between maternal mental health and breastfeeding, because we know the genetic liability was determined before the manifestation of breastfeeding. That said, this does not mean we fully understand the pathway from genetic propensity to behaviour.^42^ Second, we are also confident that this effect is not a result of residual confounding. It is therefore clear that continued support for breastfeeding, past the newborn stage, is needed to reach the WHO targets of exclusive breastfeeding to 6 months postpartum,^1^ and mothers with poor mental health are a particularly vulnerable group in need of this targeted support. Midwives routinely screen for antenatal and postnatal depression symptoms as part of routine perinatal care in the UK, therefore it is feasible to identify women who need additional support focused on breastfeeding maintenance, which could, for example, target breastfeeding challenges, managing pain and overcoming barriers to the maintenance of breastfeeding. Additionally, there is also a need for wider knowledge on how best to support women to continue to breastfeed, such as by partners, wider family members, social groups and employers.

## Supporting information

10.1192/bjo.2026.12046.sm001 Braithwaite et al. supplementary material Braithwaite et al. supplementary material

## Data Availability

ALSPAC data access is through a system of managed open access. For more information, please see http://www.bristol.ac.uk/media-library/sites/alspac/documents/researchers/data-access/ALSPAC_Access_Policy.pdf

## Supplementary material

The supplementary material is available online at https://doi.org/10.1192/bjo.2026.12046
