# Supplementary material for: Maternal genetic liability for neuroticism and breastfeeding intention, initiation and maintenance
Source: BJPsych Open. 2026 Jul 14;12(4):e188. doi: 10.1192/bjo.2026.12046 (PMC13372803; doi:10.1192/bjo.2026.12046)
Supplement: Braithwaite et al. supplementary material [file S2056472426120468sup001.docx]

**Supplementary Figure 1.** Hypothesised pathway of the impact of PGS neuroticism on breastfeeding outcomes.

**
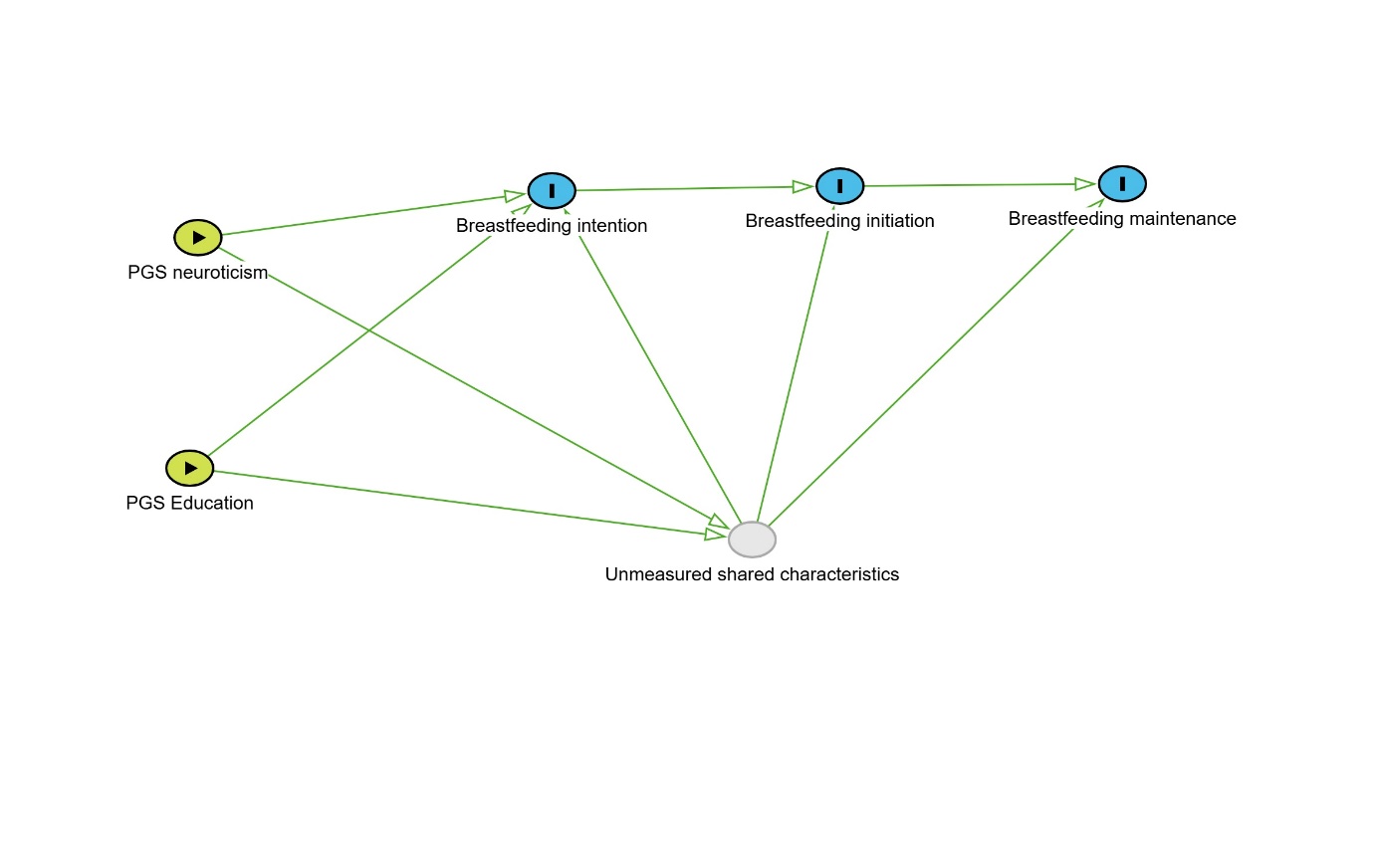
**

**Genotyping information**

ALSPAC mothers were genotyped using the Illumina human660W-quad array at Centre National de Genotypage (CNG) and genotypes were called with Illumina GenomeStudio. PLINK (v1.07) was used to carry out quality control measures on an initial set of 10,015 subjects and 557,124 directly genotyped SNPs. SNPs were removed if they displayed more than 5% missingness or a Hardy-Weinberg equilibrium P value of less than 1.0e-06. Additionally, SNPs with a minor allele frequency of less than 1% were removed. Samples were excluded if they displayed more than 5% missingness, had indeterminate X chromosome heterozygosity or extreme autosomal heterozygosity. Samples showing evidence of population stratification were identified by multidimensional scaling of genome-wide identity by state pairwise distances using the four HapMap populations as a reference, and then excluded. Cryptic relatedness was assessed using a IBD estimate of more than 0.125 which is expected to correspond to roughly 12.5% alleles shared IBD or a relatedness at the first cousin level. Related subjects that passed all other quality control thresholds were retained during subsequent phasing and imputation. 9,048 subjects and 526,688 SNPs passed these quality control filters.

3,453 ALSPAC mother and fathers and 535,478 SNPs were genotyped using the Illumina HumanCoreExome chip genotyping platforms by the ALSPAC lab and called using GenomeStudio. The resulting raw genome-wide data were subjected to standard quality control methods using PLINK (v1.07). Individuals were excluded on the basis of gender mismatches (n = 80); minimal or excessive heterozygosity (n = 64); disproportionate levels of individual missingness (>5%, n = 60) and possible contamination (n = 3). Population stratification was assessed by multidimensional scaling analysis and compared with 1000 Genomes phase 3 data and principal component analysis (n = 266); all individuals with non-European ancestry were removed. Cryptic relatedness was measured as SNP relatedness in GCTA (relatedness > 0.1, n = 69 removed). SNPs with a call rate of < 95% or evidence for violations of Hardy-Weinberg equilibrium (P < 1E-7) and those which failed GenomeStudio quality control measures were removed (n = 21,298). 6,594 duplicate SNPs were also removed. Data was phased for 3074 samples that passed QC but contained related subjects in SHAPEIT v2.r837. The following were then removed: 155,336 monomorphic SNPs, 1033 markers not in 1000 genomes, 11,842 A/T or G/C SNPs and 10 duplicate sites to give 337,732 SNPs on chromosomes 1-23. Of the 329,363 markers on chromosomes 1-22, 298,742 overlapped the reference genome. These were imputed to the 1000 genomes phase 1 version 3 using the Michigan Imputation Server. 1722 eligible partners remained after QC, exclusion of duplicate subjects and individuals who had withdrawal of consent.

| **Supplementary Table 1.** Number of SNPs included at each PT threshold for neuroticism and educational  attainment PGS | | | |
| --- | --- | --- | --- |
| PT Threshold | NEU | EA |  |
| 5.00x10^-08^ | 114 | 621 |  |
| 5.00x10^-07^ | 198 | 959 |  |
| 5.00x10^-06^ | 397 | 1596 |  |
| 5.00x10^-05^ | 910 | 2871 |  |
| 0.0005 | 2652 | 5971 |  |
| 0.005 | 9491 | 14530 |  |
| 0.05 | 39335 | 43723 |  |
| 0.5 | 159337 | 147876 |  |
| 1 | 211681 | 193674 |  |

SNPs: single nucleotide polymorphisms. PGS: Polygenic Score. PT: P-value threshold for the PRS (i.e., threshold for the number of significant SNPs from the original GWAS included into the PRS). NEU: Neuroticism. EA: Educational Attainment.

**Supplementary Table 2**. Breastfeeding variable names in ALSPAC dataset and description of variable.

| **Variable Name** | **Description** |
| --- | --- |
| C620 | Mothers reported, during pregnancy, how they intended to feed their child during the first week after birth. |
| Ka030 | Mothers reported how they fed their child in the first 24 hours after birth. |
| Ka031 | Mothers reported how they fed their child in the first week after birth. |
| Kb280 | Mothers reported whether they continued to breastfeed their child to 6 months post birth. |

**Supplementary Table 3**. Descriptive statistics of the breastfeeding variables: intention, initiation, and maintenance

|  | **Yes** | | **No** | |
| --- | --- | --- | --- | --- |
|  | **N** | **%** | **N** | **%** |
| Breastfeeding intention | 3773 | 76.83 | 1138 | 23.17 |
| Breastfeeding initiation | 3678 | 74.29 | 1273 | 25.71 |
| Breastfeeding maintenance | 1601 | 32.23 | 3366 | 67.77 |

**Supplementary Table 4**. Associations between maternal PGS for neuroticism and breastfeeding outcomes at differing PGS thresholds.

| **PGS Threshold p=0.05** | **Outcome Variable** | **Odds Ratio** | **Standard error** | **p-value** | **95% Confidence Interval** |
| --- | --- | --- | --- | --- | --- |
| N=4611 | Breastfeeding intention | 0.972 | 0.035 | 0.445 | 0.906-1.044 |
| Pseudo R^2^<0.001 |  |  |  |  |  |
| N=4611 | Breastfeeding initiation | 0.975 | 0.034 | 0.476 | 0.911-1.044 |
| Pseudo R^2=^0.001 |  |  |  |  |  |
| N=4611 | Breastfeeding maintenance | 0.864 | 0.027 | <0.001 | 0.813-918 |
| Pseudo R^2^=0.003 |  |  |  |  |  |
| **PGS Threshold p=0.02** | **Outcome Variable** | **Odds Ratio** | **Standard error** | **p-value** | **95% Confidence Interval** |
| N=4611 | Breastfeeding intention | 0.966 | 0.035 | 0.337 | 0.900-1.037 |
| Pseudo R^2^<0.001 |  |  |  |  |  |
| N=4611 | Breastfeeding initiation | 0.965 | 0.034 | 0.306 | 0.901-1.033 |
| Pseudo R^2=^0.001 |  |  |  |  |  |
| N=4611 | Breastfeeding maintenance | 0.868 | 0.027 | <0.001 | 0.817-0.923 |
| Pseudo R^2^<0.001 |  |  |  |  |  |
| **PGS Threshold p=0.005** | **Outcome Variable** | **Odds Ratio** | **Standard error** | **p-value** | **95% Confidence Interval** |
| N=4611 | Breastfeeding intention | 0.955 | 0.035 | 0.211 | 0.891-1.026 |
| Pseudo R^2^<0.001 |  |  |  |  |  |
| N=4611 | Breastfeeding initiation | 0.918 | 0.073 | 0.283 | 0.785-1.073 |
| Pseudo R^2=^0.001 |  |  |  |  |  |
| N=4611 | Breastfeeding maintenance | 0.956 | 0.035 | 0.211 | 0.891-1.025 |
| Pseudo R^2^=0.003 |  |  |  |  |  |
|  |  |  |  |  |  |
| **PGS Threshold p=0.0001** | **Outcome Variable** | **Odds Ratio** | **Standard error** | **p-value** | **95% Confidence Interval** |
| N=4611 | Breastfeeding intention | 0.991 | 0.036 | 0.819 | 0.925-1.063 |
| Pseudo R^2^<0.001 |  |  |  |  |  |
| N=4611 | Breastfeeding initiation | 0.973 | 0.034 | 0.443 | 0.908-1.043 |
| Pseudo R^2=^0.001 |  |  |  |  |  |
| N=4611 | Breastfeeding maintenance | 0.889 | 0.027 | <0.001 | 0.836-0.944 |
| Pseudo R^2^<0.003 |  |  |  |  |  |
|  |  |  |  |  |  |
| **PGS Threshold p=0.05^-7^** | **Outcome Variable** | **Odds Ratio** | **Standard error** | **p-value** | **95% Confidence Interval** |
| N=4611 | Breastfeeding intention | 0.998 | 0.036 | 0.950 | 0.929-1.071 |
| Pseudo R^2^<0.001 |  |  |  |  |  |
| N=4611 | Breastfeeding initiation | 0.918 | 0.073 | 0.283 | 0.785-1.073 |
| Pseudo R^2=^0.001 |  |  |  |  |  |
| N=4611 | Breastfeeding maintenance | 0.951 | 0.029 | 0.104 | 0.895-1.010 |
| Pseudo R^2^<0.003 |  |  |  |  |  |
